# Supplementary material for: Internal Mammary Arteries as a Model to Demonstrate Restoration of the Impaired Vasodilation in Hypertension, Using Liposomal Delivery of the CYP1B1 Inhibitor, 2,3′,4,5′-Tetramethoxystilbene
Source: Pharmaceutics. 2022 Sep 26;14(10):2046. doi: 10.3390/pharmaceutics14102046 (PMC9611804; doi:10.3390/pharmaceutics14102046)
Supplement: Supplementary file 1 [file pharmaceutics-14-02046-s001.zip › pharmaceutics-1910789-supplementary.pdf]

Internal Mammary Arteries as a Model to Demonstrate Restoration of the Impaired Vasodilation in Hypertension, Using Liposomal Delivery of the CYP1B1 Inhibitor, 2,3',4,5'-Tetramethoxystilbene

Azziza Zaabalawi[1], Lewis Renshall[2,3,4], Frances Beards[2,3,4], Adam P Lightfoot[1,8], Hans Degens[1,5], Yvonne Alexander[1], Sarin Mathew[6], Ragheb Hasan[6], Haris Bilal[6], Brigitte A Graf[7], Lynda K Harris[2,3,4], May Azzawi[1*].

1 Department of Life Sciences, Manchester Metropolitan University, Manchester, M1 5GD.
2 Division of Pharmacy and Optometry, University of Manchester, Manchester, M13 9PL.
3 Maternal & Fetal Health Research Centre, University of Manchester, Manchester, M13 9WL.
4 Manchester Academic Health Science Centre, Central Manchester University Hospitals NHS Foundation Trust, Manchester, M13 9WL.
5 Institute of Sport Science and Innovations, Lithuanian Sports University, Kaunas, 44221, Lithuania.
6 Department of Cardiothoracic Surgery, Manchester Foundation Trust, Manchester, UK.
7 Faculty of Health and Education, Manchester Metropolitan University, Manchester, M15 6BG.
8 School of Pharmacy and Biomolecular Sciences, Liverpool John Moores University, Liverpool, L3 3AP.
* Correspondence: M.Azzawi@mmu.ac.uk

Supplemental Figures

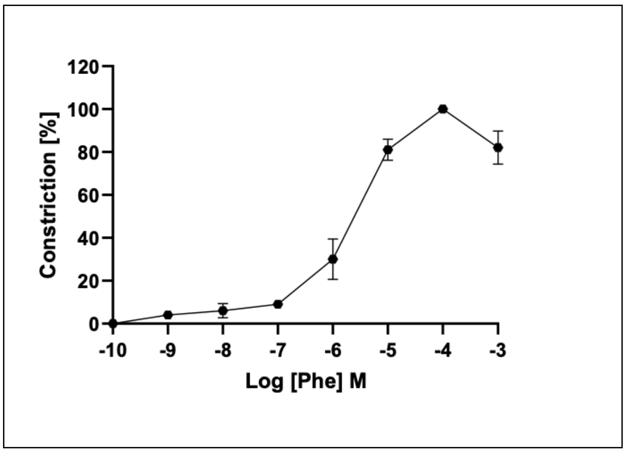

Figure S1. Constrictor responses of isolated human internal mammary arteries (IMAs) to phenylephrine (Phe). The dose-response effect of phenylephrine (Phe) (10^{-9}–10^{-3}M) in isolated IMAs from CABG patients under standard tension (2.5g). All vessels constricted to Phe in a dose-dependent manner, with sub-maximal dose identified as 10^{-5}M (n=3).

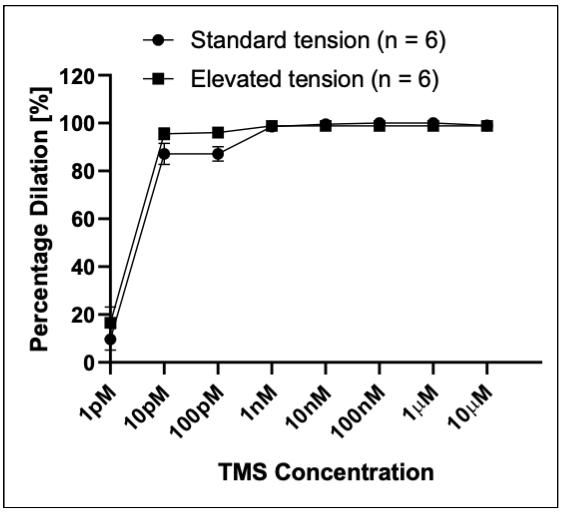

Figure S2. The effect of Tetramethoxystilbene (TMS)-loaded liposomes on vascular responses of isolated rat aortic vessels. Dilator responses to cumulative doses of TMS-loaded liposomes (1 pM – 10 μM) after standard (2 g; 1 hour) or elevated tension (4 g; 1 hour) in phenylephrine (Phe; 10^{-5} M) pre-constricted vessels. Maximal dilation was achieved at a minimal dose of 1 nM (n=6). Data were analyzed using a one-way ANOVA followed by a Dunnett's multiple comparison. Data are presented as mean ± standard error of mean.

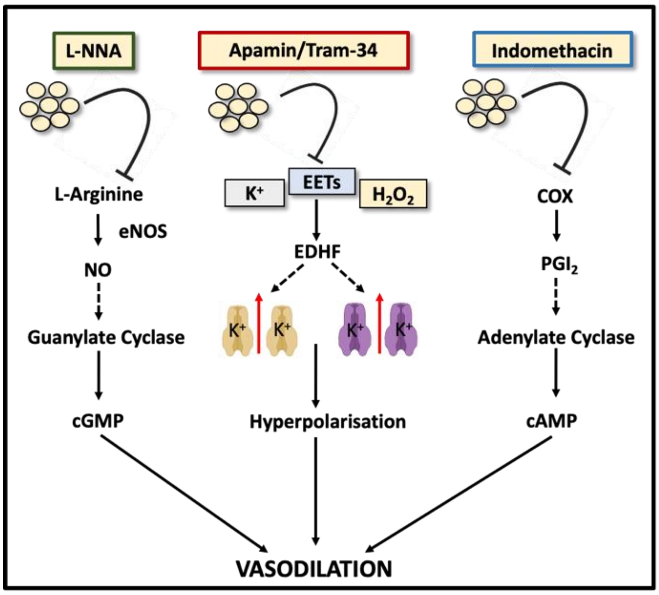

Figure S3. Schematic illustration showing pathways and targets implicated following treatment with pharmacological inhibitors used in this study.

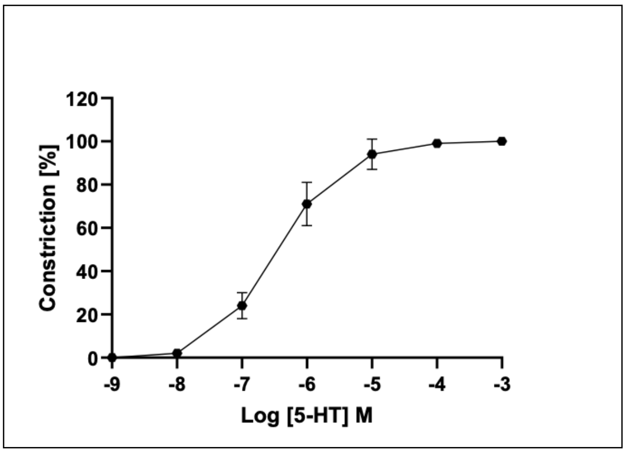

Figure S4. Constrictor responses of isolated rat coronary arteries to serotonin (5-HT). The dose-response effect of 5-HT (10^{-9}–10^{-3}M) in coronary arteries maintained under normotensive pressure (60mmHg; 1hour). All vessels constricted to 5-HT in a dose-dependentmanner, with sub-maximal dose identified as 10^{-6}M (n=4).

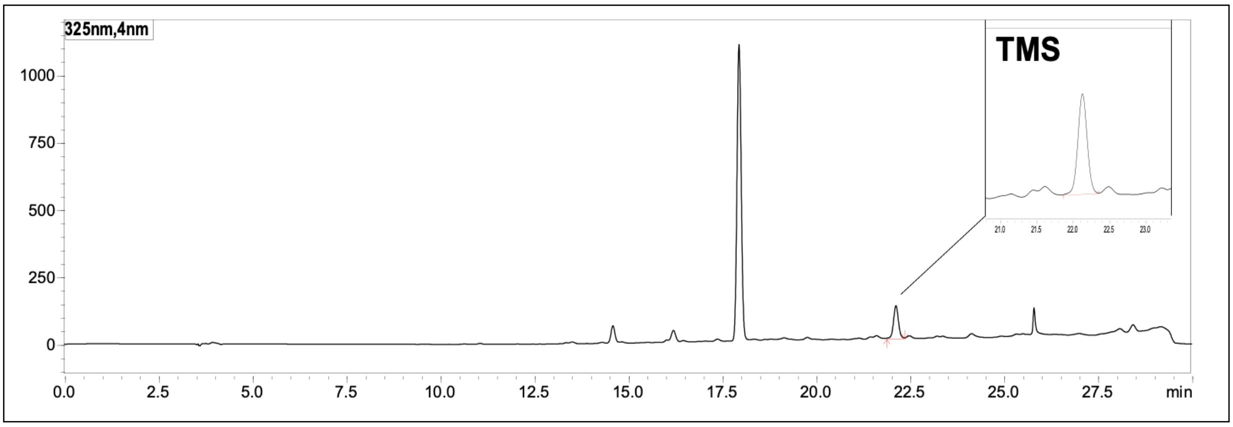

Figure S5. HPLC analysis of TMS. TMS content in TMS buffer solution after 4hours incubationwith murine coronary segments. TMS eluted after 22.18min and was identified by coelution with an authentic standard at 325nm.

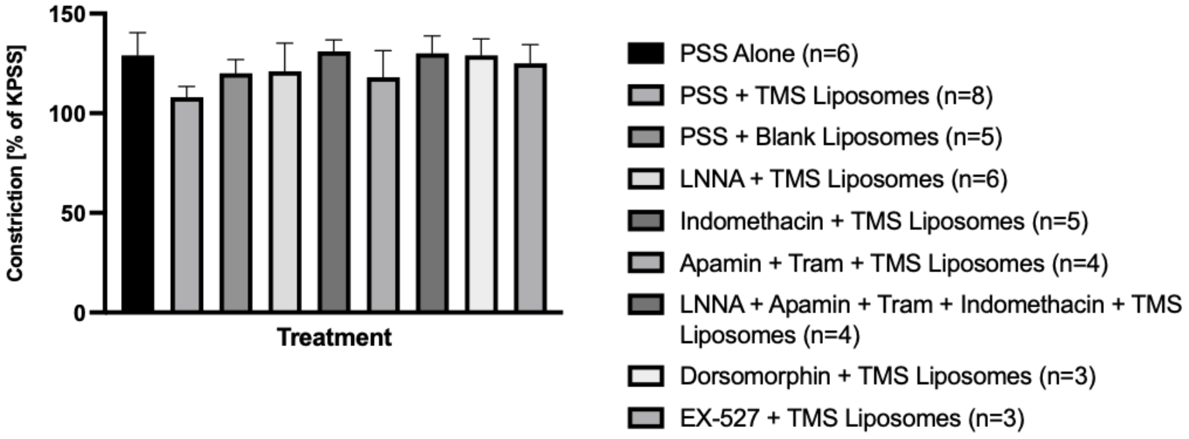

Figure S6. Pre-constrictor responses to phenylephrine (Phe) in isolated left internal mammary arteries (IMAs) from coronary artery bypass graft (CABG) patients. Treatment hadno effect on Phe (10^{-5}M) - induced vasoconstrictor responses of isolated IMAs, *ex vivo*, were similar for all vessels examined in each treatment/experimental group. One-way ANOVA followed by a Dunnett's multiple comparisons test.

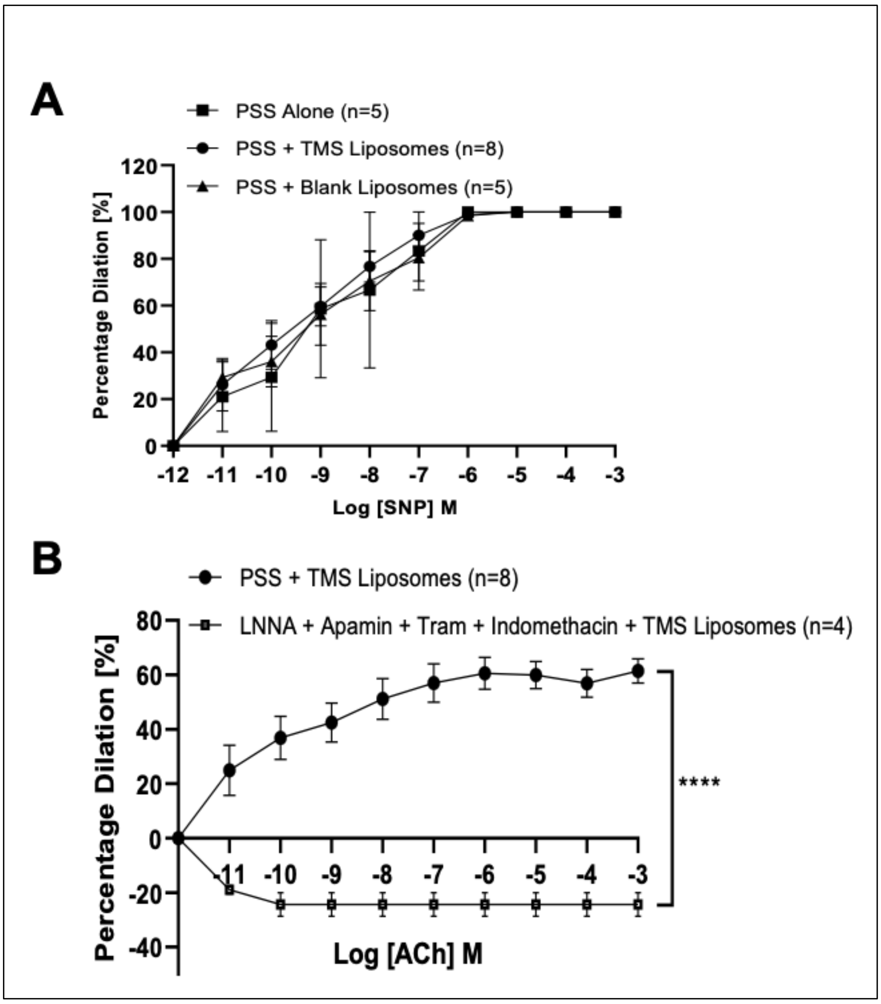

Figure S7. The influence of Tetramethoxystilbene (TMS)-loaded liposomes on vascular responses of isolated left internal mammary arteries (IMAs) from coronary artery bypass graft (CABG) patients, *ex vivo*. **(A)** Endothelial-independent sodium nitroprusside (SNP) induced responses following co-incubation with TMS-loaded liposomes. **(B)** The influence of NΩ-nitro-L-arginine (LNNA), Indomethacin, Apamin and TRAM-34 in combination on endothelial-dependent ACh-induced dilator responses following co-incubation with TMS- loaded liposomes. The area under the curve for two groups were compared using Two-way ANOVA followed by Tukey's multiple comparisons post-test. Data are presented as mean ± SEM. ****P<0.0001.

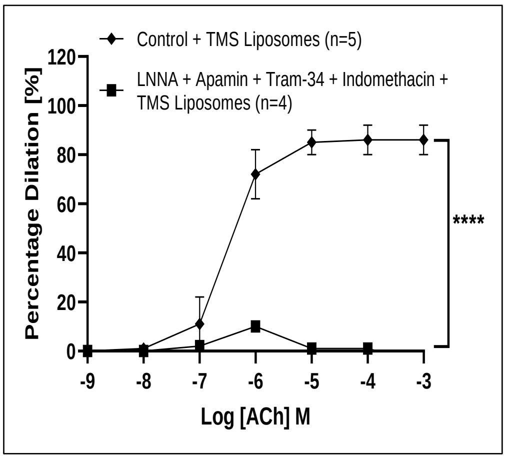

Figure S8. Characterization of dilator responses potentiated by Tetramethoxystilbene (TMS)-loaded liposomes following acute pressure elevation in isolated coronary arteries ex vivo. The influence of NΩ-nitro-L-arginine (LNNA) + Apamin and TRAM-34 + Indomethacin, in combination, on endothelium-dependent acetylcholine (ACh) responses following acute pressure elevation. The area under the curve for two groups were compared using Two-way ANOVA followed by Tukey's multiple comparisons post-test. Data are presented as mean ± SEM. ****$P < 0.0001$.
